# Rhabdomyolysis Secondary to Hypothyroidism: Report of a Case

**DOI:** 10.7759/cureus.12746

**Published:** 2021-01-17

**Authors:** Mohsen S Alshamam, Dawa O Gurung, Nso Nso, Merjona Saliaj, Adja Seitaj

**Affiliations:** 1 Department of Medicine, Icahn School of Medicine at Mount Sinai, Queens Hospital Center, NYC, USA; 2 Internal Medicine, New York City (NYC) Health + Hospitals-Queens, NYC, USA; 3 Department of Medicine, Icahn School of Medicine at Mount Sinai at Queens Hospital Center, NYC, USA; 4 Medicine, Zucker School of Medicine at Hofstra University, Hempstead, USA

**Keywords:** rhabdomyolysis, hypothyroidism, acute kidney injury, elevated ck, myoglobinuria

## Abstract

Rhabdomyolysis has many causes; however, hypothyroidism is a rare cause of such a condition. Usually, management is similar in many cases, but some exceptions do exist, especially in the case of hypothyroidism. Thus, we reviewed the literature to investigate further precipitant factors, clinical presentations, complications, management, and prognoses. We report a 19-year-old male with a history of hypothyroidism who was brought in for questionable suicidal ideation. Although asymptomatic, he was found to have an acute kidney injury (AKI). Further investigations revealed significantly elevated levels of creatine kinase (CK) and thyroid-stimulating hormone (TSH) in the setting of medication non-compliance. Management with intravenous (IV) fluids and thyroid hormone replacement resulted in an improvement in AKI and CK levels.

## Introduction

Rhabdomyolysis is a rare but dangerous complication of hypothyroidism. Even though many patients present with the classic signs and symptoms of hypothyroidism, in rare cases, rhabdomyolysis can only be the presenting illness. Though medication non-adherence in cases of established hypothyroidism has been reported, suboptimal dosing of thyroid hormone also can cause such manifestation. Very often, precipitating factors accelerate the development of rhabdomyolysis in hypothyroidism, but rhabdomyolysis can happen in the absence of such factors. Even though symptoms of rhabdomyolysis can be seen in the majority of cases, the absence of such symptoms should not be used to exclude the diagnosis. A high index of suspicion to drive a prompt diagnosis and treatment should always be instituted. Such patients are found to have an excellent response to early management with fluids and thyroid hormone replacement.

## Case presentation

**Figure 1:**
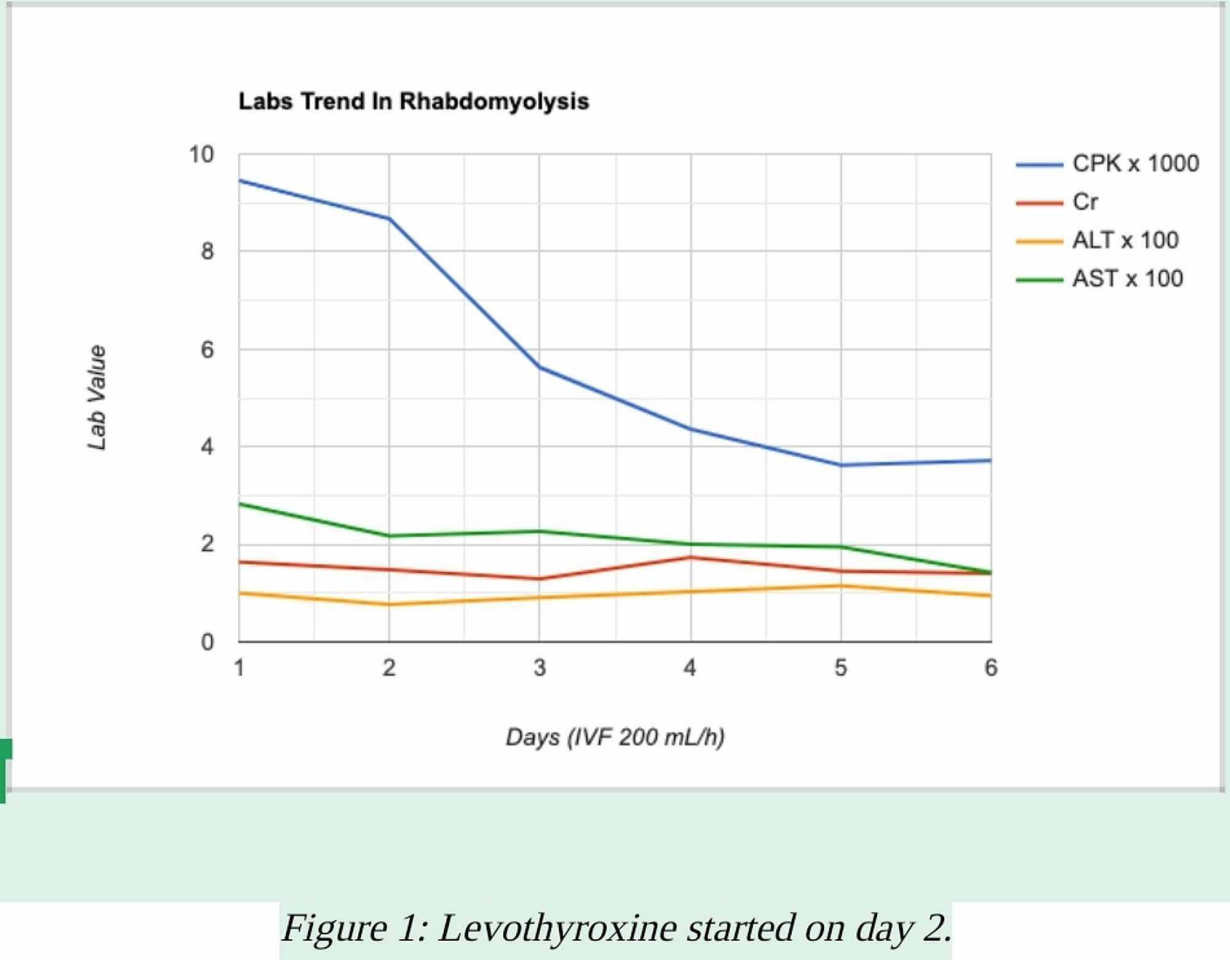
Lab trends before and after levothyroxine initiation

A 19-year-old male with a past medical history of congenital hypothyroidism without goiter, polysubstance abuse (alcohol, marijuana, psilocybin “mushroom”) was brought in by emergency medical services (EMS) after he was found in a maintenance complex of Long Island Railroad. Upon history-taking, the patient denied any suicidal or homicidal ideation but was found to be delusional. Further evaluation did not reveal any acute complaints. The patient denied any recent alcohol or drug use for the last week. He also denied any fever, chills, chest pain, shortness of breath, nausea, vomiting, abdominal pain, diarrhea, dysuria, hematuria, muscle pain, or weakness. He was on levothyroxine 150 mcg, which he stopped taking a month ago because it was making him anxious. The physical exam was unremarkable with normal vital signs and negative for bruising, hematoma, paresthesia, myalgias, rigidity, or obvious trauma. Laboratory workup revealed: creatinine (Cr) 1.6 (mg/dL), calcium (Ca) 10.4 (mg/dL), creatinine kinase (CK) 9344 (U/L), lactate dehydrogenase (LDH) 621 (U/L), thyroid-stimulating hormone (TSH) 838 (uIU/mL), free triiodothyronine (T3) 0.39 (pg/mL), urinalysis (UA) positive for moderate blood and red blood cells (RBCs) 10-20, aspartate transaminase (AST) 283 (U/L), alanine transaminase (ALT) 100 (U/L), and total bilirubin 1.8 (mg/dL). Labs were normal/negative for ethanol, aminosalicylic acid (ASA), acetaminophen, five-panel drug screen, C-reactive protein (CRP), and D-dimer. A 10-panel drug screen was positive only for tetrahydrocannabinol (THC) 14 (ng/mL). A computed tomography (CT) of the head without contrast and a chest X-ray did not show any acute pathology. Three liters (3L) bolus of intravenous (IV) fluids in the form of normal saline were initially started, and the patient was continued on IV fluids at a rate of 200 mL/hr. He had minimal improvement after seven liters of IV fluids; however, after levothyroxine 125 mcg was resumed, a day after admission, the patient started to show satisfying improvement. Given the patient was delusional, he was seen by the psychiatry department and was diagnosed with a brief psychotic disorder, psychotic disorder secondary to substance abuse, or psychotic disorder due to an underlying medical condition. He was started on risperidone 0.5 mg twice daily, a day after admission, which he took for two days then declined thereafter. The patient’s lab markers continued to normalize (Figure 1), and his psychological state improved to baseline. He was discharged home six days after admission on levothyroxine 125 mcg daily with plans to follow up as an outpatient.

## Discussion

The term hypothyroidism, generally, refers to the decreased function of the thyroid gland, which can be primary or secondary. The prevalence of hypothyroidism is estimated to be somewhere between 4% and 12% of the total population, comprising both the overt and subclinical aspects of the disease [1]. The symptoms of hypothyroidism are widely distributed across many systems and include fatigue, weight gain, cold intolerance, constipation, depression, weakness, and muscle pain. Rhabdomyolysis is a rare but dreadful complication of hypothyroidism [2].

Rhabdomyolysis refers to the breakdown/destruction of skeletal muscle cells. Causes of rhabdomyolysis are many, including drugs, toxins, infections, trauma, metabolic factors, genetic defects, exercise, and dehydration [2-3]. Multiple steps in cellular injury culminate in intracellular calcium release, resulting in the hyperactivity of proteases and proteolytic enzymes and oxygen-free radical generation, ending in myofilament degradation and injury. Furthermore, membrane injury leads to intracellular content release (i.e., potassium, calcium, phosphate, uric acid, and myoglobin) into the bloodstream, complicating the condition [4]. However, the exact etiology of rhabdomyolysis in hypothyroidism still remains unclear [2].

Symptoms of rhabdomyolysis, though not always present, include muscle pain, weakness, and myoglobinuria; however, the absence of such symptoms should not drive the basis of excluding hypothyroidism as a cause of rhabdomyolysis [5]. Even though precipitants such as drugs (i.e., statins and antibiotics), toxins, infections, and exercise were reported [2,6-7], patients can have no precipitating factors prior to presentation [4,6,8-12].

Furthermore, though rare, rhabdomyolysis can be the only presenting illness of undiagnosed hypothyroidism [6,8,10-12], or in an established case where there is medication non-compliance [2,9]. A prompt diagnosis requires a high index of suspicion, whether in the absence of significant medical history or in a history of hypothyroidism with medications non-adherence, to avoid the detrimental complications of rhabdomyolysis, such as acute renal failure or serious metabolic derangements necessitating the initiation of hemodialysis [7,9,12] or worsening medical condition needing a higher level of care and mechanical ventilation [12]. More importantly, prompt intervention, tackling the hypothyroid aspect of the condition, should be initiated as soon as possible; after which the desired response may only be reached [2,6-9,11-12]. Also, in patients with established hypothyroidism, where medication non-adherence was not an issue, an increase in thyroxine dose may be necessary to overcome such complications [4]. The overall prognosis of such complications after the initiation of thyroid replacement therapy is excellent [6-9,11-12].

In our patient, other causes of rhabdomyolysis were unlikely and ruled out given his negative screening. Neuroleptic malignant syndrome (NMS) was also unlikely given the absence of typical symptoms of NMS (i.e., fever, diaphoresis, or muscle rigidity) and no known use of any inciting agent (risperidone was used only two days after admission). Additionally, the significant improvement in laboratory parameters and his clinical and psychological states after initiating thyroid replacement therapy argues in favor of hypothyroidism as the sole cause of his rhabdomyolysis. Rhabdomyolysis usually responds to IV fluid management, which is the mainstay of therapy; however, approximately 7000 mL of IV fluids showed mild improvement in CK and Cr levels (Figure 1) prior to Levothyroxine initiation. Therefore, thyroid hormone supplementation is important as early as possible to avoid the grave complications of rhabdomyolysis and achieve a desirable response in such cases.

## Conclusions

Given the vast majority of causes of rhabdomyolysis, a clinician should always place hypothyroidism as a potential cause on his differential. With that said, the condition has a good and favorable response once the hypothyroidism is addressed, which prevents the cumbersome complications of rhabdomyolysis, without which such response will not be achieved. In established hypothyroidism, medication non-adherence should be suspected and addressed appropriately; whereas, in cases where compliance is not an issue, an increase in thyroxine dose should be considered.
